# Valorization Challenges to Almond Residues: Phytochemical Composition and Functional Application

**DOI:** 10.3390/molecules22101774

**Published:** 2017-10-20

**Authors:** Iva Prgomet, Berta Gonçalves, Raúl Domínguez-Perles, Núria Pascual-Seva, Ana I. R. N. A. Barros

**Affiliations:** 1 Centre for the Research and Technology of Agro-Environmental and Biological Sciences, CITAB, University of Trás-os-Montes e Alto Douro, UTAD, Quinta de Prados, 5000-801 Vila Real, Portugal; bertag@utad.pt (B.G.); rdperles@utad.pt (R.D.-P.); abarros@utad.pt (A.I.R.N.A.B.); 2 Department of Plant Production, Universitat Politècnica de València, 46022 València, Spain; nupasse@prv.upv.es

**Keywords:** *Prunus dulcis*, by-products, phenolic compounds, biological activity, functional application

## Abstract

Almond is characterized by its high nutritional value; although information reported so far mainly concerns edible kernel. Even though the nutritional and commercial relevance of the almond is restricted to almond meat; to date; increasing attention has been paid to other parts of this fruit (skin; shell; and hull); considered by-products that are scarcely characterized and exploited regarding their properties as valuable sources of bioactive compounds (mainly represented by phenolic acids and flavonoids). This lack of proper valorization procedures entails the continuation of the application of traditional procedures to almond residues that nowadays are mainly addressed to livestock feed and energy production. In this sense; data available on the physicochemical and phytochemical composition of almond meat and its related residues suggest promising applications; and allow one to envisage new uses as functional ingredients towards value-added foods and feeds; as well as a source of bioactive phytochemicals to be included in cosmetic formulations. This objective has prompted investigators working in the field to evaluate their functional properties and biological activity. This approach has provided interesting information concerning the capacity of polyphenolic extracts of almond by-products to prevent degenerative diseases linked to oxidative stress and inflammation in human tissues and cells; in the frame of diverse pathophysiological situations. Hence; this review deals with gathering data available in the scientific literature on the phytochemical composition and bioactivity of almond by-products as well as on their bioactivity so as to promote their functional application.

## 1. Introduction

The almond tree (*Prunus dulcis* (Mill.) D. A. Webb, *Prunus amygdalus* Batch or *Amygdalus communis* L.) is one of the most popular nut trees grown worldwide under arid conditions due to its resistance and capacity against water shortage and irrigation deficit. The almond tree belongs to the family Rosaceae and is placed under the genus *Prunus*, together with peach, plum, apricot, cherry, and nectarine—a group of fruits collectively known as “stone fruits”, i.e., fruits with a stony endocarp. Nowadays, the biggest almond producer is the USA, accounting for 80.0% of the world production, which reached 1.11 × 10^8^ kg (on the kernel basis) in the 2015/2016 season, being the most produced nut in the world [1]. Along with the kernel, around 0.8–1.7 M tonnes of almond shells are discarded annually [2], while the amount of almond hulls is estimated to exceed 6 M tonnes [3].

The edible part of almonds, the kernel, is a seed with two large cotyledons [4]. During fruit development, kernel is protected by the shell and the hull, whilst during fruit ripening, the hull starts to split, and once dried, the kernel easily separates from the shell. In terms of yield, average kernel weight is the most important parameter, as this involves the most valuable nutritional composition. Generally, kernel weight increases continuously until maturity, although different growing conditions and several environmental stressors such as water and temperature can cause abnormal filling [4], making productions less appreciable by the market. In the last several years, market demand for the consumption of almonds as edible nuts and as an ingredient in manufactured food products has significantly increased due to the physico-chemical, nutritional, and sensorial features of these fruits [5]. Indeed, almond kernels are consumed raw worldwide, cooked or dry-roasted, sliced or whole, blanched (without the skin) or unblanched (with the skin). It is extensively used in bakery and confectionery products, and in food preparation in general [6,7].

As mentioned before, the growing interest in almonds is based on their properties as a reliable source of nutrients, such as lipids, proteins, minerals, and dietary fiber, wherein lipids provide around 50.0% of the kernel weight (49.9 g of total lipids/100 g, according to US Department of Agriculture (USDA) database), and most unsaturated fatty acids (oleic, about 70.0% and linoleic, about 20.0%). The protein content ranges from 16.0 to 22.0% of kernel weight amongst different varieties (21.2 g of protein/100 g, according to USDA database). The amino acids are mainly bound to the almond protein amandin, whilst the content of free amino acids is negligible. On the other hand, the content of dietary fiber of almond kernels ranges from 10.8 to 13.5% (12.5 g of total dietary fiber/100 g, according to USDA database), being constituted by pectin, cellulose, and xyloglucans, as the most important components. Regarding vitamins, almonds are rich in lipid-soluble (E type, with its major homologue α-tocopherol and minor ones γ-tocopherol, β-tocopherol, and α-tocotrienol) and in small amounts of water-soluble (B type, B1, B2, B3, B5, B6, B7, B9) vitamins. The most abundant minerals in almond kernels are calcium, iron, magnesium, sodium, zinc, manganese, and selenium, and they are an especially valuable dietary source of potassium and phosphorus (733 and 481 mg/100 g, respectively, according to USDA database) [8]. Amarowicz et al. (2005) reported high concentrations of phenolic compounds in almond seeds, including benzoic and cinnamic acid derivates (vanillic, caffeic, *p*-coumaric, and ferulic acids), flavonols (quercetin, kaempferol, and isorhamnetin), anthocyanidins (delfinidin and cyanidin), and procyanidins (B2 and B3) [9].

Although almond consumption was traditionally perceived as unhealthy due to its high fat content, recent studies have evidenced the beneficial effects enclosed to the frequent consumption of nuts due to the capacity of their bioactive nutrients and non-nutrients to lower the plasma levels of low-density lipoprotein cholesterol (LDL-C) and the incidence and severity of cardiovascular disorders [10,11,12,13,14]. Indeed, results obtained so far suggest that the presence of different mechanisms are responsible for beneficial effects significantly correlated with the chemical and phytochemical composition of almonds. The bioactivity of the almond bioactives is closely linked to the bioavailability of these molecules. Grassby et al. (2017) recently reported the importance of the cell-wall barrier mechanism in the regulation of lipid bioaccessibility from different sizes of almond particles, and highlighted the crucial role played by the food matrix in the bioaccessibility of nutrients [15]. Berryman et al. (2011) tentatively identified nutrients contributing to LDL-C decreases in plasma that have been associated with almonds consumption. Therefore, among the most relevant contributors, they highlight fatty acids, phytosterols, dietary fibers, proteins, amino acid arginin, and micronutrients, such as α-tocopherol [14].

Food quality is an essential characteristic that involves physico-chemical and compositional issues. In this sense, the International Organization for Standardization (ISO) defines quality as “the totality of features and characteristics of a product or service that bear on its ability to satisfy stated or implied needs” [16]. Product quality is determined by distinct characteristics or properties such as sensorial properties (appearance, texture, aroma, and taste), nutritive values, chemical constituents, mechanical properties, functional properties, and type and percentage of present defects [17]. Almond kernel quality is mainly determined by its moisture, oil content, fatty acid content, sugar, fiber, and protein content [18,19]. In addition, concerning bioactive non-nutrients, the influence of phenolic compounds and their radical scavenging activity on almond fruit quality and human health have been widely reported [11,20,21,22].

All these factors have boosted the production and consumption of almonds; however, kernel quality and yields are not the only issues concerning producers, food manufacturers, and scientists. Hence, it is important to notice that, besides edible nuts (kernel, meat, or seed) directly addressed to fresh consumption, these fruits, before processing, also consists of several materials that are not consumed as such, including brown skin (seed coat), shell (hardened endocarp), and hull (flesh but thin mesocarp, green shell cover). Given the lack of proper characterizations of these materials, in the last several years, there has been a special focus on the bioactive phytochemicals present in almond by-products, as well as their application and valorization. In this sense, many authors have reported a higher phenolic content and radical scavenging power in almond by-products than in the kernel itself [6,23,24]. In this regard, for instance, Wijeratne et al. (2006) found that the total phenolic content of hull and skin ethanolic extracts, compared with whole seed extracts, were nine- and ten-fold higher, respectively [6]. In the same study, the valuable antioxidant capacity of the above mentioned extracts was also noticed by monitoring the inhibition of human LDL oxidation, the inhibition of DNA scission, and metal ion chelation activities [6]. Polyphenolic skin extracts were demonstrated to have an efficient capacity to inhibit copper-induced oxidation of LDL-C at 50 ppm, while whole seed and hull extracts reached the same level of efficiency at 200 ppm. Hull extracts completely arrested peroxyl radical-induced DNA scission at 50 ppm, and seed and skin extracts had a similar efficiency at 100 ppm. Despite these differences, it has to be stressed that the extracts obtained of all three materials exert excellent metal ion chelating efficacy [6].

Taking into consideration the state of the art concerning the composition and applicability of almond by-products, the present review deals critically with the agro-economical relevance of almond by-products based on their physico-chemical and biological properties.

## 2. Almond By-Products

**Figure 1:**
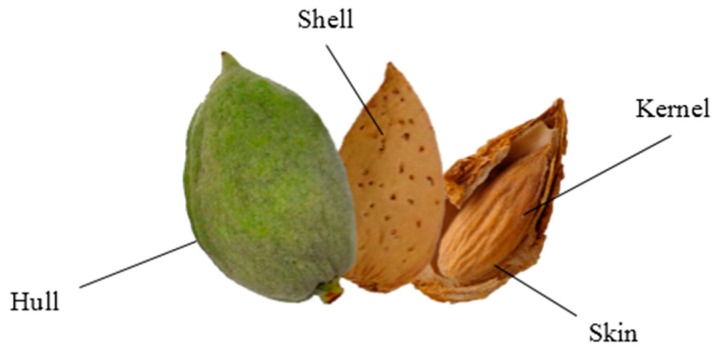
Parts of almond fruit.

Almond production generates large amounts of by-products. While the nutritional and commercial relevance of almonds is restricted to the kernel, almond by-products (Figure 1), constituted by hull, shell, and skin, remain underexplored, and further information on their physico-chemical features, composition, and optimization of extraction procedures is required to determine sustainable and competitive exploitation alternatives that are compatible with current commercial uses.

**Figure 2:**
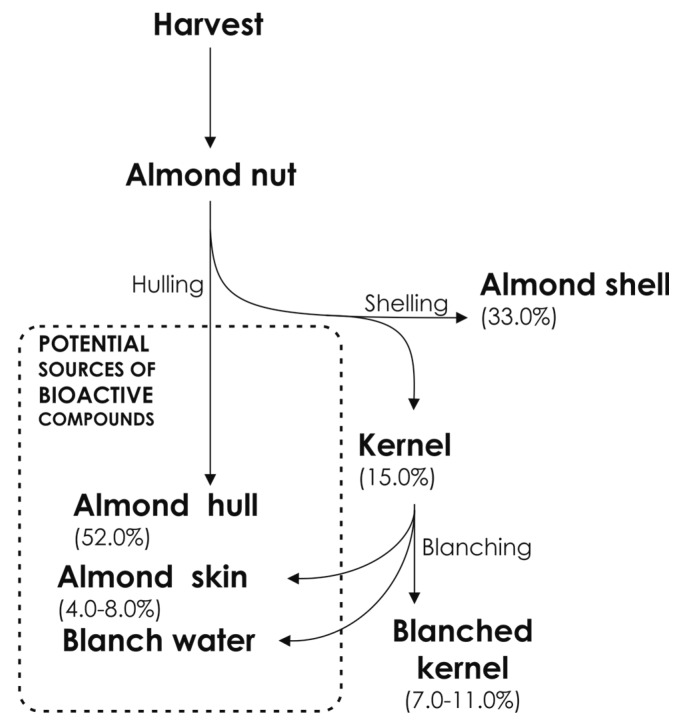
Process of obtaining almond by-products in the almond industry.

Considering the physico-chemical properties of the diverse solid wastes from almond processing, the heaviest material is represented by the green hull, with an average of 52.0% of the total fresh weight, whilst the shell and kernel (including skin) account for around 33.0% and 15.0% of the total fresh weight, respectively [25]. During the industrial process of removing skin (in the blanching process), two further by-products, in addition to the shell and the hull, are produced: blanched skin (4.0–8.0% of the total shelled almond fresh weight) and blanch water. Thus, 70.0–85.0% of the whole almond fruits constitute residues of this agro-food activity at the end of processing (Figure 2). Currently, almond by-products (mainly hulls and shells) are mostly used as cattle feed, and bedding. Hulling and shelling are expensive operations, whilst the returns obtained from them do not cover the processing investments, which jeopardize the sustainability and competitiveness of this industrial activity [26]. On the other hand, the physico-chemical properties and the composition of these materials suggest possible alternative applications and have prompted researchers to envisage different solutions that should be considered, such as the recovery of valuable bio-based compounds and their use as natural food preservation additives and dietary/nutraceutical supplements.

### 2.1. Almond Skin

Almond skin (seed coat) can be removed and discarded after blanching. The proximate composition of almond skin includes total dietary fiber (47.5 and 45.1 g/100 g of blanched and natural skin, respectively), soluble dietary fiber (2.7 and 3.8 g/100 g blanched and natural skin, respectively), lipids (22.2 and 24.2 g/100 g of blanched and natural skin, respectively), and proteins (12.8 and 10.3 g/100 g of blanched and natural skin, respectively) [27]. In addition to the basic chemical composition, although skin represents only about 4.0% of the total almond weight [23] and has been associated with a very low economic value, a number of recent studies have reported that it contains about 60.0–80.0% of total phenolic compounds existing in the nut. It is important to state that phenolic compounds present in almond skin are responsible to a significant extent for the high radical scavenging activity capacity demonstrated for such materials [28,29,30,31,32], and possibly for the beneficial effects associated with almond consumption.

### 2.2. Almond Shell

Almond endocarp is known as shell, composed of compact arrangements of lignocellulosic sclereid cells. It is primarily composed of cellulose (ranging from 29.8 to 50.7%), hemicellulose (from 19.3 to 29.0%), and lignin (from 20.4 to 50.7%) [33]. The hardness of a shell is associated with the total amount of lignin formed during the nut development [4], morphology, fiber content, and the outer shell adherence [33].

### 2.3. Almond Hull

The almond hull is the thin mesocarp or green shell cover, which forms 35.0–62.0% of total almond fresh weight [34]. Its thickness and weight differ significantly among varieties; in some of them, it is thin and dry and provides a small portion of the whole fruit, while other varieties have thick and fleshy hulls, providing a large proportion of the fruit weight [4]. The hull characteristics condition the fruit removal from the tree. In addition, the features of hulls have a direct influence on drying after harvest as well as the efficiency of hull removal [4]. The nutritional composition of almond hulls depends not only on the almond variety to a great extent, but also on environmental factors and agronomical management that contribute strongly to the final physico-chemical and phytochemical properties of almond kernel and its by-products. Hence, the sugar content in almond hulls reportedly ranges from 18.0 to 30.0%, protein content varies from 2.1 to 8.8%, and crude fiber ranges from 10.0 to 24.9%. Acid detergent fiber varies from 20.6 to 35.2%, neutral detergent fiber from 10.0 to 15.0%, cellulose from 20.6 to 35.2%, and crude lignin ranges from 7.5 to 15.6%. Depending on the harvest method, ashes can vary from 7.0 to 8.3%, but can be even higher than 9.0%. In these cases they are classified as “almond hulls and dirt” [35].

## 3. Phytochemical Composition of Almond By-Products and Their Radical Scavenging Power

Plants are rich sources of natural antioxidants including tocopherols, vitamin C, carotenoids, and phenolic compounds [36]. These compounds have been promoted as relevant molecules against oxidative stress because of their radical scavenging activity.

Although free radicals and other reactive oxygen species are continuously formed in the human body, an imbalance between oxidants (free radicals) and antioxidants, in favor of oxidants, has been related to oxidative stress, which is implicated in the pathophysiology of several human diseases.

**Figure 3:**
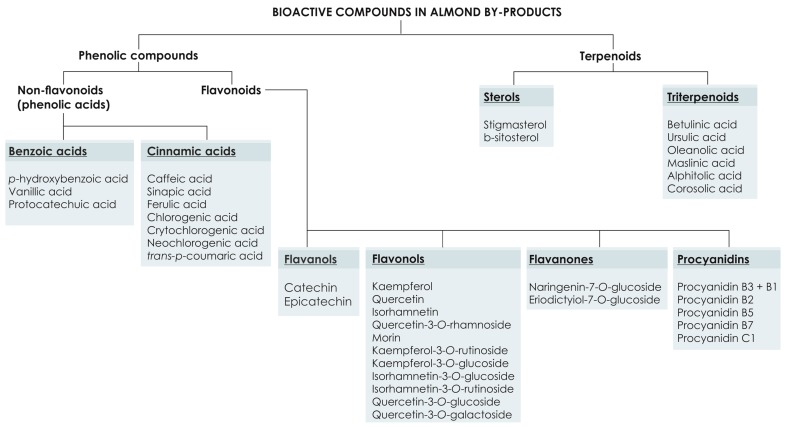
Bioactive compounds in almond by-products.

Resorting to their functional features, natural antioxidants have been promoted as molecules that increase the shelf life of products in the food industry, preferably used instead of synthetic antioxidants (such as BHA and BHT). In this view, the presence of the bioactive compounds in almond and its by-products (Figure 3) and their biological activities in almond hulls [3,37,38,39,40,41], almond skin [28,29,30,31,32], almond shells [7,42], and blanch water [23,32,43] have been studied, and is the main aspect discussed further in this manuscript.

### 3.1. Phenolic Compounds

**Figure 4:**
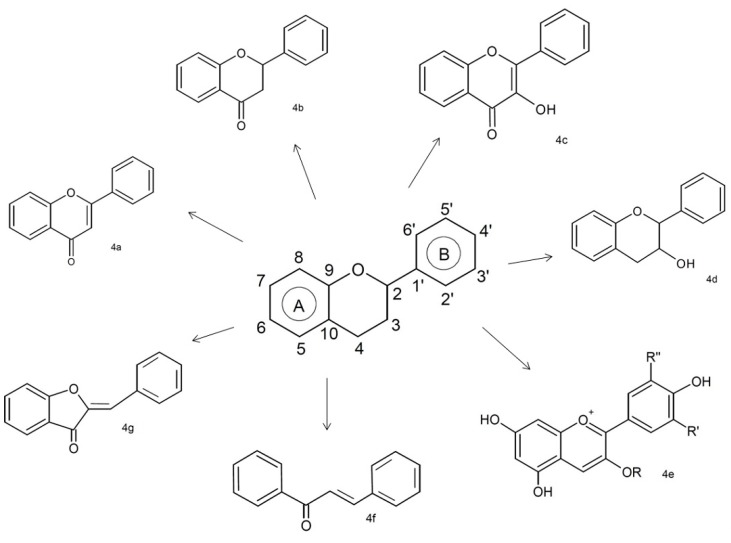
Structures of flavonoids class.

**Figure 5:**
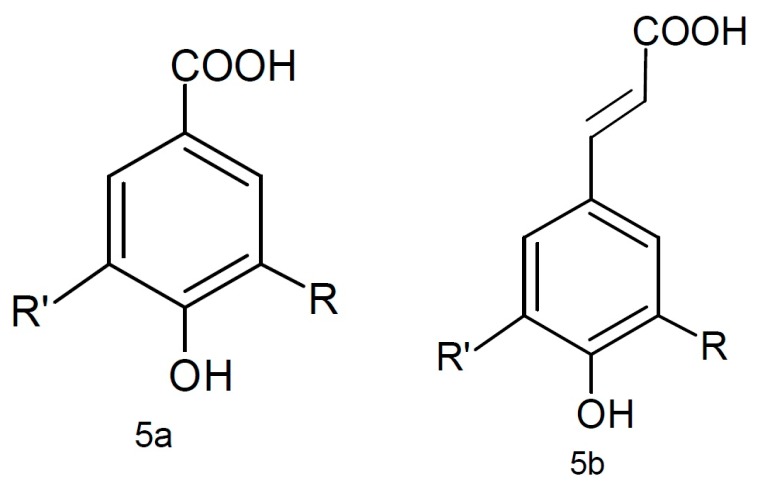
Non-flavonoids: benzoic (5a) and cinnamic (5b) acids.

Phenolic compounds are the major group of phytochemicals in almonds. These classes of compounds are featured by including at least one aromatic ring, with one or more hydroxyl groups attached, and are classified as flavonoid (Figure 4) and non-flavonoid compounds (Figure 5). The flavonoids class (Figure 4) is composed of flavones (4a), flavanones (4b), flavonols (4c), flavanols (4d), anthocyanins (4e), chalcones (4f), and aurones (4g), and the non-flavonoids (phenolic acids) mainly include benzoic and cinnamic acids (5a and 5b, respectively) [44,45].

#### 3.1.1. Flavonoids

Flavonoids are water-soluble pigments, sharing a common structure of two aromatic rings bound together by three carbon atoms forming an oxygenated heterocycle [46]. This type of phenolic compounds is synthesized through the phenylpropanoid pathway, where phenylalanine is transformed to 4-coumaroyl-CoA, which then enters the flavonoid biosynthesis pathway. Biological functions of flavonoids in plants are related to defense against UV radiation and pathogen infection, pollen fertility, nodulation [47], symbiotic nitrogen fixation, and floral pigmentation, acting also as chemical messengers, cell cycle inhibitors, and physiological regulators [48]. In plants, flavonoids can occur in a form of aglycones or glycosides, more often found as the latter one [49].

The composition of flavonoids in plants is influenced by several factors such as variety, geographical origin, agronomical practices, ripeness stage, processing, storage, environmental conditions, exposure to pests and diseases, environmental conditions, and UV radiation [23].

#### 3.1.2. Phenolic Acids

Phenolic acids are secondary metabolites characterized by hydroxylated aromatic ring(s). Hydroxybenzoic and hydroxycinnamic acids are produced via shikimic acid through the general phenylpropanoid pathway. However, despite the long term during which the chemical features and biological functions of phenolic acids have been studied, to date, the data are still scarce, with gaps of information regarding their capacity to modulate biological processes in plants. Anyway, the biological functions of phenolic acids have been related to protein synthesis, photosynthesis, and nutrient uptake [50]. In plants, phenolic acids can be found in different forms, such as aglycones, glycosides, esters, and bound complexes [49].

### 3.2. Phenolic Composition of Almond Skin

In the industry, almond skin is obtained by blanching or roasting. Upon these processes, the entire almond is subjected to high temperatures (around 100 °C for blanching and up to 200 °C for roasting) to obtain skinless almond kernels. Even though the almond skin represents just around 4.0% of the total almond weight, 60.0–80.0% of the almond phenolic compounds are distributed within the almond skin [23,51]. However, thermal processes in the food industry may affect the stability of polyphenols and promote the degradation and loss of bioactive compounds [52]. For instance, albeit subjected to lower temperatures compared to industrial roasting, almond skin polyphenols are easily lost by hot water blanching and demonstrate a lower phenolic content [53]. Furthermore, results from one study suggested that the majority of the phenolic compounds in the kernel itself does not consist of flavonoids [23]. Eleven out of 19 phenolic compounds identified in almond kernels, skin, and blanch water (Table 1) were found only in the skin, whilst 95.0% of the individual flavonoids identified were also present in the skin [23]. This situation also points out the role of flavonoids as phytoalexins, secondary metabolites that plants synthesize for self-defense, protecting the skin from different stresses.

Sang et al. (2002) isolated nine phenolic compounds that were identified as flavonol glycosides, flavanone glycoside, and flavanol and benzoic acid derivatives (Table 1) [28]. Upon the determination of the radical scavenging activity using the 1,1-diphenyl-2-picryl-hydrazyl (DPPH) test, the strong radical scavenging activity of catechin, protocatechuic acid, vanillic acid, and *p*-hydroxybenzoic acid, as well as of all identified flavonol glycosides, except kaempferol-3-*O*-α-l-rhamnopyranosyl-(1→6)-β-d-glucopyranoside, which exhibited very weak DPPH radical scavenging activity, was demonstrated. The basis of the DPPH assay is the scavenging of DPPH radicals by antioxidant molecules [59,60] since the radical compound is stable and does not need to be generated [60]. Thus, this is one of the methods more widely used for determining the antioxidant activity of different food matrices, although frequently its results are complemented by those provided by other methods evaluating molecules with different polarity or the reduction power. This approach is justified by the existence of numerous methods to determine the radical scavenging activity, while their use depends on some factors such as the nature of the sample, the mechanism of generation of target molecules, and the end-product determination, among others [61]. Many studies have been focused on determining the antioxidant activity of bioactive compounds in almond skin by the radical scavenging methods DPPH [28,31,32,62] and ABTS [56], the ferric reducing antioxidant power (FRAP) assay [56,62,63], and the oxygen radical absorbance capacity (ORAC) assay [30,31,53,56,62]. Most of them provides data supporting almond skin as a matrix containing a phytochemical composition with highly valuable antioxidant activity. The radical scavenging activity of extracts of almond skin is mainly due to its content of phenolic compounds. Once the interest of these bioactive compounds is determined, the polyphenolic extracts of almond skin are further characterized via high-performance liquid chromatography (HPLC) coupled with UV/VIS detection and mass spectrometry. Matrix-assisted laser desorption/ionization time-of-flight mass spectrometry (MALDI-TOF/MS) was used by Frison-Norrie and Sporns (2002) [58] to detect and quantify four flavonol glycosides—isorhamnetin rutinoside, isorhamnetin glucoside, kaempferol rutinoside, and kaempferol glucoside (Table 1)—in the almond skin. In a further study, these authors assessed skins of 16 different almond varieties using the same MALDI-TOF/MS technique as previously [64], which allowed for the detection of the same four flavonol glycosides, with isorhamnetin rutinoside being the most plentiful one, in all the studied almond varieties. However, MALDI-TOF/MS cannot distinguish between some compounds, such as ishorhamnetin-3-galactoside and isorhamnetin-3-glucoside, as they have identical molecular weights. Furthermore, Arraez-Roman et al. (2010) have recently developed and compared two rapid methods for the separation and characterization of phenolic compounds in almond skin extracts: CE-ESI-TOF-MS and HPLC-ESI-TOF-MS [55]. Using a 35 min gradient, the method described by Arraez-Roman allowed for the determination of nine (9) compounds under optimum CE-ESI-TOF-MS conditions (Table 1), while, using the HPLC-ESI-TOF-MS method, a 9 min gradient allowed for the identification of 23 compounds corresponding to phenolic acids and flavonoids (Table 1). In addition, Bolling et al. (2009) developed a routine, conventional, noncapillary LC-MS method for quantifying polyphenols from almond skin, which provided the tentative identification and quantification of 16 flavonoids and two phenolic acids (Table 1), and concentrations ranging from 1.4 to 2.2 mg polyphenols/g almond skin were found [57]. However, as one of the objectives was to reduce the analysis duration, some of the compounds were not properly differentiated, such as isorhamnetin-3-*O*-glucoside and isorhamnetin-3-*O*-galactoside, and quercetin-3-*O*-galactoside and quercetin-3-*O*-glucoside, when using this method. Bolling (2017) recently assessed in his review all methods of analysis used for identification and quantification of almond polyphenols, mainly based on the kernel but also on its by-products [65].

Almond skin can be removed in the industry by blanching (and then peeling) or roasting, processes that can change the polyphenolic composition of almond skin. Garrido et al. (2008) have concluded, when comparing different industrial processing of removing skin from the kernel, that the most efficient industrial process of removing almond skin, maintaining its phenolic composition almost intact and with high antioxidant activity, is roasting, followed by blanching and oven drying [53]. In this study, from three different almond varieties, during distinct harvest years and each industrial process, the occurrence of 31 phenolic compounds corresponding to flavanols (in the highest percentage, 33.0–56.0% of the total phenolics), flavonol glycosides, hydroxybenzoic acids and aldehydes, flavonol aglycones, flavanone glycosides, flavanone aglycones, hydroxycinnamic acids, and dihydroflavonol aglycones was observed (Table 1).

As blanching is the most commonly used process in the industry for skin removal, Mandalari et al. (2010) analyzed differences between natural almond skin and blanched almond skin regarding their phenolic composition [32]. Combinations of flavonols, flavanols, hydroxybenzoic acids, and flavanones were identified in almond skin powders and in industrial blanch water. The most representative flavonoids present in almond skin were catechin, epicatechin, kaempferol, and isorhamnetin (as 3-*O*-rutinoside or 3-*O*-glucoside). In the same study, the total phenolic content in almond skin was 3474.1 ± 239.8 mg GAE/100 g of fresh skin, while for blanched almond skin the total phenolic content was 278.9 ± 12.0 mg GAE/100 g of fresh skin. As expected, natural almond skin showed the highest radical scavenging activity, followed by blanched almond skin and blanch water. However, blanched almond skin presented excellent radical scavenging activity in the DPPH assay, which can be explained by the biodegradation of bioactive compounds at high temperatures, generating other products with antioxidant properties. Similarly, Smeriglio et al. (2016) characterized the phenolic composition and antioxidant activity of industrial by-products of Avola almonds (blanched skin and blanch water), and compared the results with the values retrieved for natural almond skin [56]. Hence, resorting to RP-HPLC-DAD, 21 flavanons, flavonols, flavanols, and phenolic acids were identified (Table 1), with naringenin being the most abundant compound, followed by kaempferol-3-*O*-rutinoside, kaempferol-3-*O*-glucoside, kaempferol, and eriodictyol-7-*O*-glucoside. From this work, it was retrieved that the blanching process causes a significant decrease in the polyphenol content in almond skin, more than 60.0%, as the total phenolic content expressed in mg GAE/100 g of fresh weight was 703.0 ± 15.9 for natural skin, 313.8 ± 2.3 for blanched skin, and 73.9 ± 0.5 for blanch water. It is obvious that the blanching process can lower the phenolic content of almond skin, and that derived from this differential polyphenolic composition, natural almond skin displays a higher antioxidant power. However, technological requirements make the development of this process in the almond industry mandatory. It is required that knowledge of the development of new value-added products for these residues is gained, as this knowledge would contribute to the reduction of their environmental impact and the enhancement in the sustainability and competitiveness of the almond industry.

In addition to the previously described processes, raw almonds are often pasteurized (to prevent *Salmonella* poisoning) and can be stored for long periods of time before being sold. Bolling et al. (2010) compared the influence of roasting, pasteurization, and storage on phenolic content and antioxidant activity [63]. Indeed, initially, it was expected that these processes decrease the phenolic content and radical scavenging activity. Skin from roasted processed almond showed a significant decrease in total phenolic content and antioxidant activity, while phenolic acids remained unchanged. Pasteurization did not have any significant effect on the phenolic content, while long-term storage (15 months) had a positive effect on the concentration of total phenolic, phenolic acids, and radical scavenging activity, indicating that storage is a dynamic process upon which the physical structure of the food matrix considered could be altered, allowing a more efficient diffusion of the phenolic compounds [63].

Almond skin polyphenols are present as flavonoids in aglycone and glycoside forms, with aglycones exhibiting a higher efficiency regarding radical scavenging activity than the glycoside species [6]. Major phenolic compounds found in almond skin are flavanols and flavonol glycosides, followed by non-flavonoids [30]. Within flavanols, the most abundant compounds identified are catechin and epicatechin, while isorhamnetin-3-*O*-rutinoside and kaempferol-3-*O*-rutinoside are the major flavonol glycosides present in almond skin [30,66]. Milbury et al. (2006) reported that isorhamnetin (in the form 3-*O*-glucoside or 3-*O*-rutinoside), representing around 70.0% of the total, is the predominant flavonoid in most of the varieties assessed so far, with 97.1 ± 2.3% of compounds originating from skin and 2.9 ± 2.3% from kernel [23]. However, during the blanching process around 69.0% of isorhamnetin-3-*O*-glucoside and isorhamnetin-3-*O*-rutinoside are extracted to the blanch water [23]. Garrido et al. (2008) stated that catechin, which represents 10.0–23.0% of the phenolics identified, and isorhamnetin-3-*O*-rutinoside (6.8–17%) are the most abundant phenolics in almond skin in their study [53]. The presence and the quantity of each phenolic compound in almond skin might depend on factors such as the industrial process to which it was subjected, the storage conditions, and the extraction method, among others. Predominant phenolic acids (non-flavonoids) in many foods of plant origin are derivatives of hydroxybenzoic and hydroxycinnamic acids. Wijeratne et al. (2006) identified derivatives of cinnamic acids in almonds (with, caffeic, ferulic, *p*-coumaric, and sinaptic acids as major ones in almond skin) (Table 1) [24], whilst others identified hydroxybenzoic acids (protocatechuic, *p*-hydroxybenzoic, and vanillic acid) [23,28,57] and hydroxycinnamic acids (chlorogenic acid) [30,53] as most abundant in almond skin.

### 3.3. Phenolic Composition of Almond Blanch Water

Blanching is a process consisting in the removal of almond skin by scalding almonds in hot water for a short period of time, in effect removing most flavonoids and other phenols from almond skin. Milbury et al. (2006) noticed that, in the blanching process, kaempferol and quercetin do not leach to the water [23], while Mandalari et al. (2010) confirmed that quercetin-3-*O*-galactoside and isorhamnetin are two additional compounds that are not extracted to water during blanching [32]. This fact has been attributed to their poor water solubility. In addition, Hughey et al. (2012) did not detect quercetin-3-*O*-rutinoside in blanch water [67], as well as nonpolar aglycones, such as isorhamnetin, quercetin, and kaempferol. To test the hypothesis of the constraint represented by the “limited water solubility” to the leaching of phenolic compounds into the blanching water, blanch water precipitate was analyzed. This approach demonstrated that nonpolar aglycones (isorhamnetin and kaempferol), partially insoluble glycosides (quercetin glycoside, kaempferol glycoside, rutinoside, isorhamnetin glycoside, rutinoside, and naringenin glucoside), and even more water-soluble catechins were present in blanch water precipitate, indicating that these compounds leach from the skin into blanch water, dissolve until their solubility limit, and then precipitate [67].

Furthermore, Mandalari et al. (2013) studied the antioxidant effect of blanch water [43], determining the polyphenolic content of blanch water extracts, the level of proanthocyanidins, and the vanillin index. The total phenolic content, the antioxidant activity, and the radical scavenging activity were evaluated with different in vitro tests (Folin–Ciocalteu method, DPPH radical scavenging activity, β-carotene bleaching test, reducing power test, UV-induced peroxidation in liposomal membranes). Resorting to these determinations, the total phenolic content expressed as mg of gallic acid equivalents (GAE) per g of the blanch water extract was 90.28 ± 5.47, similar to the concentrations reported by Milbury et al. (2006) that described values ranging between 50.3 and 153.9 mg GAE released from blanching 100 g of fresh natural almonds [23]. The most representative compounds identified in blanch water extracts were naringenin-7-*O*-glucoside and kaempferol-7-*O*-rutinoside, followed by catechin. The valuable antiradical activity of the extract was demonstrated within the DPPH and reducing power tests.

Kinetics of the almond skin separation via hot water submersion need to be understood to achieve it more efficiently, save energy, and possibly lower production costs [68]. Fisklements and Barrett (2014) [68] have reported that skin separation rates at 90 °C and 100 °C were not significantly different, while the rate decreased rapidly below 90 °C. At the same time, the relevance of these findings lies in understanding the distribution of polyphenols in blanch water and almond blanched skin, as well as those factors with a relevant impact on this distribution. On the other hand, according to Hughey et al. (2012), temperature and time increased the amount of phenolic compounds extracted into water [67]. These authors reported that, during the first 2 min and under a temperature of almost 100 °C (that is, time and temperature used in the industry for blanching), approximately 73.0% of phenolics leach from almond skin into water, rising to 90.0% after 10 min.

The presence of value-added antioxidant compounds in the blanch water extracts reveals the possibility of further utilization and valorization of the blanch water by the nutraceutical and pharmaceutical industries.

### 3.4. Phenolic Composition of Almond Hulls

Almond hulls are a major by-product in almond production, being mostly used as cattle feed. The phenolic constituents and the radical scavenging activity, as well as the bioactivities of this almond green cover, have been identified and quantified. Sang et al. (2002) isolated protocatechuic acid, catechin, ursolic acid, and prenylated benzoic acid derivatives from almond hulls (Table 1) [37]. Protocatechuic acid is one of the major benzoic acid derivatives with strong antioxidant power, and catechin is the most widely distributed flavonoid in edible plants, with a high antioxidant activity and an inhibitory effect on numerous enzymes.

Takeoka and Dao (2003) extracted almond hulls using methanol and identified present compounds with reversed phase HPLC with diode array detection [54]. In the extract, three hydroxycinnamic acids were identified: chlorogenic acid (5-*O*-caffeoylquinic acid), cryptoclorogenic acid (4-*O*-caffeoylquinic acid), and neochlorogenic acid (3-*O*-caffeoylquinic acid) in the ratio 79.5:14.8:5.7 (Table 1), chlorogenic acid appearing as the most abundant phenolic acid in the almond hull extract. In addition, extracts were tested for their ability to inhibit the oxidation of methyl linoleate at 40 °C, and the result showed that, at the same concentration, almond hull extracts present a higher antioxidant activity than α-tocopherol. At higher concentrations, almond hull extracts presented increased antioxidant activity, with similar values to chlorogenic acid and morin standards at the same concentration, showing that almond hulls are potential sources of these dietary antioxidants.

In addition, Rubilar et al. (2007) analyzed ethanol extracts and fractions (organic/water fraction) of almond hulls and grape pomace for antioxidant power and their phenolic profiles were determined by HPLC-MS [40]. In the almond hull extracts, chromatographic peaks evidenced the occurrence of benzoic and cinnamic acid derivatives, with a small presence of flavan-3-ols, while an additional presence of epicatechin and glycosilated flavonols have been identified in the fractions. The antiradical activity was assessed using both DPPH and TBARS assays, and results showed a stronger antiradical activity of grape pomace than almond hulls.

Another study on antioxidant properties of almond hulls has been performed by Barreira et al. (2010), evaluating it through a range of chemical and biochemical assays on different almond varieties in Portugal [41]. Samples were extracted using water, as the aim of the study was to obtain a clean extract that can be used as an additive due to the synergistic effects of the phytochemicals present in fruits and vegetables and the benefits of the complex mixture in whole foods. All assayed by-products (almond hulls, chestnut leaves, and skins) revealed good antioxidant properties with very low EC50 values.

Wijeratne et al. (2006) identified eight phenolic compounds from almond extracts of almond whole seed, brown skin, and hulls [6]. High-performance liquid chromatography (HPLC) analysis allowed for a description of the presence of flavonols and flavonol glycosides as major flavonoids in all three extracts (Table 1). In another study, antioxidant and antiradical activity of phenolic extracts of almond hulls and shells, obtained from different genotypes were compared by Sfahlan et al. (2009) [7]. The authors selected 18 genotypes from diverse Iran provinces and determined total phenolic content using Folin–Ciocalteu method, reducing power, and scavenging capacity for radicals nitrite, hydrogen peroxide, and superoxide. Within various genotypes significant differences were found in the phenolic content of hulls and shells, as well as on the percentage of radical scavenging capacity. However, among different genotypes, the total phenolic content of almond hulls extract was higher than the one of the correspondent shells.

## 4. Biological Activities of Almond By-Products

Phytochemicals are compounds known for their biological effects including anticancer, antiviral, and anti-inflammatory activities, among others, demonstrated upon a number of experimental procedures conducted with in vitro and in vivo (in animals and humans) models [69,70,71].

Almond skin, hulls, and blanch water have been shown to have antimicrobial and antibacterial potential [56,72] as well as anti-inflammatory, photoprotective, and prebiotic effects [27,43,73,74,75], antitumor properties [39], and antiviral activity [76]. Based on these biological properties, by-products from the almond industry have been promoted as useful functional ingredients useful for the control of oxidative process due to their strong antioxidant/antiradical activity [30,41] and more specifically, enhancing effect on the resistance of human LDL to oxidation [29].

Almond skin polyphenols are bioavailable in the upper gastrointestinal tract and potentially available for absorption during digestion [66,77]. Hence, blanched skin obtained during the industrial process developed to obtain marketable almond kernels constitutes a potential source of bioactive compounds. Furthermore, the antimicrobial potential of almond skin refers to this by-product as a potential source of natural antimicrobials. Mandalari et al. (2010) evaluated the antimicrobial properties of fractions derived from almond skin, against Gram-positive and Gram-negative bacteria, and the yeast [72]. Almond skin fractions (both natural and blanched) exhibited antibacterial activity against two Gram-positive bacteria (*Listeria monocytogenes* and *Staphylococcus aureus*) in the range 250–500 µg/mL, and natural skin against the Gram-negative bacterium *Salmonella enterica* [72]. In addition, Smeriglio et al. (2016) tested extracts of natural and blanched skin and blanch water [56]. In this work, all three extracts were active against Gram-positive bacteria (*Bacillus subtilis*, *Enterocossus durans*, *Enterocossus hirae*, *Listeria monocytogenes*, *Straphylococcus aureus*, *Straphylococcus epidermidis*, and *Streptococcus mutans*), except for the blanched skin extract regarding *B. subtilis*, *E. hirae*, and *S. mutans*. However, the activity in both studies was shown to be bacteriostatic and never bactericidal [58,70].

Moreover, almond skin has been proven to have prebiotic effects. Mandalari et al. (2010) evaluated the potential prebiotic effect of natural and blanched almond skin using a full model of the gastrointestinal tract, including in vitro gastric and duodenal digestion that was followed by colonic fermentation using fecal bacterial cultures [27]. More recently, this group demonstrated that the bioaccessibility of almond skin polyphenols is significantly affected by the type of used food matrix, such as home-made biscuits, crisp-bread, and full-fat milk [78]. Additionally, Liu et al. (2014) investigated the prebiotic effects of almond and almond skin intake in healthy humans and analyzed fecal samples for microbiota composition and selected indicators of microbial activity [75]. In all of these studies, results indicated the stimulation effects of almond skin as typical prebiotic effects, probably due to a high content of dietary fiber that is resistant to digestive enzymes, so it passes to the large intestines where it interacts with microbiota to induce gut health [75]. However, dietary polyphenols present in almond skin may confer positive gut health benefits as well, as they have the potential to alter gut microecology and positively affect the number of beneficial microbiota [79]. Phenolic compounds could possibly bind protein present in the food matrix and, even though bioaccessibility of each antioxidant differs significantly, the potential synergistic effect between polyphenols is likely to affect their bioactivity and influence glycoprotein transporters across the mucosal epithelium [78].

In addition to this antimicrobial power, blanch water and almond skin demonstrated a photoprotective effect. Sachdeva and Katyal (2011) evaluated almond skin extract on the capacity of its extract to confer human skin protection against solar UV radiation, as well as on antiaging activity developed by inducing UV-B oxidative stress in mice and applying the topical treatment of almond skin extract formulation 2 h prior to UV exposure [74]. In another study, the formulation of 2.0% of blanch water extract was used as a topical photoprotective agent in vivo, i.e., on human volunteers that were suffering from the skin erythema induced by acute UV-B exposure [43], obtaining an inhibition of erythema by 50.5%. These studies retrieved evidence supporting that these two almond by-products had a positive effect against photooxidative damage.

Natural and blanched polyphenols-rich almond skin extracts exhibited an antiviral activity blocking herpes simplex virus type 1 (HSV-1) infection in a recent study by Bisignano et al. (2017). In this study, natural skin extracts blocked the production and spreading of HSV-1 particles to other, neighboring cells, indicating that the most abundant compound in such extract, quercetin, was capable of inhibiting HSV-1, supporting the development of new functional products with virucidal properties aimed at the prevention of HSV-1 infections [76].

When considering the potential application of almond hulls, it should be highlighted that this constitutes a potential source of terpenoids such as betulinic, oleanolic, and ursolic acids, known for their antitumor and anti-HIV properties. Amico et al. (2006) have subjected almond hull extracts (fractions of crude extract extracted with ethyl acetate) to the MTT (3-(4,5-dimethylthiazol-2-yl)-2,5-diphenyltetrazolium bromide) bioassay with MCF-7 human breast cancer cells, and from the most active fraction isolated several compounds [39]. The most active compound of all was proved to be betulinic acid, which showed, interestingly, significantly higher antiproliferative activity against MCF-7 cells than a well-known anticancer agent for clinical use, 5-fluorouracil.

## 5. Functional Application of Almond By-Products

Functional applications of almond by-products are resumed in Table 2, with a focus on the industries where it is applied and on their uses.

Additionally, extracts from almond residues (specifically almond hull, dried blanched/roasted almond skin and blanch water), considering their antioxidant and bioactive activity, have great potential to be used as natural food preservation additives, dietary/nutraceutical supplements or/and in the pharmaceutical and cosmetic industry. Indeed, the technological capacity already existing may allow for the recycling and valorizing of these materials as valuable ingredients through the recovery of bioactive compounds suitable for the manufacturing process towards the development of new value-added products. Galanakis (2012) has recently reviewed the technology advances gained in the last few decade to recover value-added components from food waste by conventional and commercialized alternatives [93]. In this work, five sequential common recovery stages (macroscopic pre-treatment, macro and micro molecules separation, extraction, isolation and purification, and product formation) by different technologies (conventional and emerging) that lead to the recovery of high value-added components from food waste, applied from the source to the final product, were stated. The main steps to the encapsulation of the target phenolic compounds are extraction (or removal) of specific micro-molecules, purification, and finally encapsulation.

The optimization of the extraction conditions constitutes a critical procedure for the full valorization of agro-food by-products, since this first step may influence the final usage of the extracts obtained, due to their impact on the presence of toxic substances or on the relative abundance of the individual compounds. There are many factors in almond production that can influence successful extraction with desirable characteristics, including preharvest and climatic conditions, orchard location, agricultural practices to the genetic (varietal) background, and harvest time. In addition, industrial processing (blanching and roasting, among others), storage conditions, plant material quality, sample preparation and the extraction technique used may have a great influence on the overall extract quality as well. All of these factors might be crucial for obtaining high quality extracts, with high phenolic yield and beneficial biological functions.

Solvent extraction is a conventionally established technique for the recovery of phenolics; however, due to its pure selectivity and to ensure the identity and safety of the target compounds, additional purification is usually required. Commonly used conventional techniques include adsorption [94], chromatographic techniques [95], nanofiltration, and electrodialysis [96], whose efficiency proportionally affects the cost, while emerging ones, such as membrane capsules and ion-exchange chromatography, offer lower operating and capital investment costs [93].

The active ingredient obtained are then encapsulated, providing significant protection against oxidation and degradation, leading to enhanced shelf life to the target bioactive compounds. Recently, Munin and Levy (2011) have classified encapsulation processes applied to polyphenols into physical, physico-chemical, chemical method, and other connecting stabilization methods, confirming encapsulation as an interesting mean to boost the biological activity of polyphenolic compounds/extracts [97]. The spray-drying method was highlighted as the most common technique used to encapsulate polyphenols. However, to the best of our knowledge, so far there are no studies reporting encapsulation of polyphenols from almond by-products, despite their being a valuable source of bioactive compounds.

In addition to these uses, and given the chemical and phytochemical composition of almond shells, it can be successfully used for other purposes, such as mulch, natural colorant, and/or growing media, as explained below.

### 5.1. Almond Shell as Mulch

Appropriate mulching is a key tool in organic production systems providing several advantages, namely protecting soil from erosion, preventing weed growth, improving soil moisture, and plant establishment and growth, increasing soil nutrition, and reducing diseases and pesticide use [83]. Traditionally, the reuse of forestry and agricultural by-products as mulching materials has been of great interest for growers. Residual organic materials from crops planted nearby could be a great source for mulch, reducing the carbon footprint of the whole production chain. In a 10-year long study, Lopez et al. (2014) analyzed the long-term effects of the successive application of almond shell as mulch for organically grown avocado trees [84]. Almond shells were applied three times—in 2002, 2007, and 2012—and the treatment was compared to the conventional (using mineral fertilizers and herbicides) no-tillage treatment. In this work, similar or higher yield, parallel to lower costs in comparison to the conventional management were obtained. In addition, the reduced mineralization and high surface biological activity and the increased soil organic carbon were a positive consequence, indicating almond shells mulch to be ideal in avocado production. It is expected that almond shell mulch would have similar effects on other crops, which might be especially important for the organic farmers.

### 5.2. Almond Shell as Natural Colorant

Ecofriendly natural dyes have lately been of great interest as a result of the worldwide growing consciousness of using recycled, reused, and ecologically acceptable materials. Ismal and Yildirim (2012) [91] used almond shells to extract natural dye for dyeing of the wool, using different mordants (iron (II) sulfate, copper (II) sulfate, and potassium dichromate). Mordants are essential components of the natural dyeing process, which fix the dye to the substrate by combining it with the dye pigment and forming the insoluble compound, obtaining varied color shades and fastness properties [92]. Depending on the type of mordants, with the same dyestuff, different colors and shades on the dyed material can be obtained. It was observed that wool fibers can be dyed in different colors and depth of shades with the almond shell. Iron (II) sulfate showed the best results in light fastness, and even control (without mordants) resulted in good wash fastness. In this study, almond shell was considered as a new alternative natural colorant source. Continuing, Ismal et al. (2014) studied even more ecological approach, replacing the mordants with biomordants (biological natural material having metal ion(s), tannins, and coming mostly from vegetal sources) such as powder of valex, pomegranate rind, rosemary, and thuja leaves in the dyeing with the almond shell [92]. Results retrieved showed biomordands as an alternative method to help in the reduced use of toxic metal salt mordants.

### 5.3. Almond Shell and Hull as Growing Media

Another ecologically-friendly approach using almond shells and hulls has evidenced them as a good alternative to widely used materials. Production of the soilless crops is increasing and there is an interest to find suitable substitutes for traditionally used substrates. Urrestarazu et al. (2005) used 100.0% pure almond shell as growing media and evaluated it as substrate for soilless production [85]. The effects of volume and texture were evaluated and compared with rockwool in terms of yield and quality characteristics of fruits in tomato and melon culture. No limitations were found comparing to rockwool in relation to fertigation parameters, yield, and water uptake, for tomato and melon crops. Therefore, almond shells seemed to be an acceptable growing media for soilless cultures, representing an ecologically friendly alternative to the rockwool. Furthermore, Urrestarazu et al. (2008) conducted another study, upon which almond shells were evaluated as growing media for long-term soilless production, in order to be competitive [86]. The results suggested that commercial almond shells seem to be acceptable growing media for vegetable production after at least 530 days of reutilization. Study of the use of both almond shells and hulls as substrates for sweet pepper cultivation and their effect on fruit yield, size, and mineral content was conducted by Valverde et al. (2013), demonstrating that the almond shell showed better results than the hull, indicating a viable alternative to soil for pepper cultivation [87].

## 6. Conclusions

For producers, enhanced agricultural productivity, as well as staying competitive, is relevant, but at the same time, the importance also lies in the improvement of the environmental impact by giving an added value to waste accumulated in agriculture. The almond industry, as the most substantial nut industry, plays an important role in generating bio-waste, since 70.0–85.0% of the whole almond fruit are its by-products—hulls, shells, and skin, which becomes a by-product after the process of blanching and generates another by-product: blanch water. These parts have been evaluated for their phenolic composition and bioactivities related to present phytochemicals, compounds strongly dependent on a myriad of factors, such as processing and storage conditions, among others. Benefits that each almond by-product possess that can be further valorized vary depending on almond by-product. Skin, hulls, and blanch water represent sources of bioactive compounds with strong antioxidant, antimicrobial, prebiotic, antitumor, antiviral, and photoprotective properties, while almond shells present properties to be used in other areas, such as agriculture, energy production, and as livestock bedding.. Even though all residues of the almond industry might represent a valuable source of raw materials for cosmetic, pharmaceutical, food, and agricultural industries, their valorization needs further examination and commitment for the development of novel time-saving procedures to be implemented to the sectorial industries. Finally, providing innovative products from almond residues could contribute to the reduction of environmental impact and provide added value to the overall almond production.

## Figures and Tables

**Table 1:** Phenolic compounds present in almond by-products.

Phenolic Class	Compound	Radicals						Almond Fruit Parts	Content	References
***Phenolic acids***										
**Cinnamic acids**		R_1_	R_2_	R_3_	R_4_					
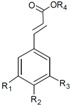	Caffeic acid	H	OH	OH	H			Hull	Traces	[24]
								Skin	Traces	[24]
	Sinapic acid	CH_3_O	OH	CH_3_O	H			Hull	9.92 ^a^	[24]
								Skin	9.51 ^a^	[24]
	Ferulic acid	H	OH	CH_3_O	H			Hull	2.71 ^a^	[24]
								Skin	2.19 ^a^	[24]
	Chlorogenic acid	H	OH	OH	C_7_H_11_O_5_			Hull	42.52 ^c^	[54]
								Skin	3.12–10.6 ^a^ 1.76–3.77 ^a^ + 9571.26 ^b^ 62.21 ^e^	[30] [53] [55] [32] [56]
	Crytochlorogenic acid	H	OH	OH	C_7_H_11_O_5_			Hull	7.9 ^c^	[54]
	Neochlorogenic acid	H	OH	OH	C_7_H_11_O_5_			Hull	3.04 ^c^	[54]
	*trans*-*p*-coumaric acid	H	OH	H	H			Hull	1.34 ^a^	[24]
								Skin	4.55 ^a^ 0.725 ^a^ 1.06 ^a^ + 367.71 ^b^ 31.87 ^e^	[24] [30] [53] [55] [32] [56]
***Phenolic acids***										
**Benzoic acids**		R_1_	R_2_	R_3_						
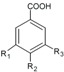	*p*-hydroxybenzoic acid	H	OH	H				Skin	+ 17.5 ^d^ 5.33–6.88 ^a^ 3.08–7.18 ^a^ + + 6401.20 ^b^ 127.17 ^e^	[28] [23] [30] [53] [57] [55] [32] [56]
	Vanillic acid	H	OH	CH_3_O				Skin	+ 18.2 ^d^ 7.65–14.5 ^a^ 11.1–19.2 ^a^ + 5805.23 ^b^ 425.15 ^e^	[28] [23] [30] [53] [55] [32] [56]
	Protocatechuic acid	H	OH	OH				Hull	+ +	[37] [6]
								Skin	+ 17.6 ^d^ + 14.5–32.0 ^a^ 6.69–17.2 ^a^ + + 1541.67 ^b^ 285.12 ^e^	[28] [23] [6] [30] [53] [57] [55] [32] [56]
***Flavonoids***										
**Flavanols**		R_3_	R_5_	R_7_	R_3’_	R_4’_	R_5’_			
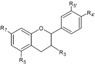	Catechin	OH	OH	OH	H	OH	OH	Hull	+	[37]
								Skin	+ 35.7 ^d^ 36.40–90.10 ^a^ 20.1–38.3 ^a^ + + 15,569.00 ^b^ 183.67 ^e^	[28] [23] [30] [53] [57] [55] [32] [56]
	Epicatechin	OH	OH	OH	H	OH	OH	Skin	33.9 ^d^ 14.8–36.6 ^a^ 7.17–26.5 ^a^ + + 10,955.6 ^b^ 52.4 ^e^	[23] [30] [53] [57] [55] [32] [56]
***Flavonoids***										
**Flavonols**		R_3_	R_5_	R_7_	R_4’_	R_5’_	R_6’_			
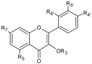	Quercetin	H	OH	OH	OH	OH	H	Hull	+	[6]
								Skin	+ 100 ^d^ 1.43–1.78 ^a^ 1.02–4.89 ^a^ + + 214.3 ^b^ 802.2 ^e^	[6] [23] [30] [53] [57] [55] [32] [56]
***Flavonoids***										
**Flavonols**		R_3_	R_5_	R_7_	R_4’_	R_5’_	R_6’_			
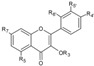	Quercetin-3-*O*-glucoside	C_6_H_11_O_5_	OH	OH	O^−^	OH	H	Skin	+ 24.5 ^d^ 1.33–2.41 ^a^ 1.71 ^a^ + 166.9 ^e^	[28] [23] [30] [53] [55] [56]
	Quercetin-3-*O*-galactoside	C_6_H_11_O_5_	OH	OH	O^−^	OH	H	Skin	+ 41.4 ^d^ + 60.6 ^e^	[28] [23] [57] [56]
	Quercetin-3-*O*-rhamnoside	C_6_H_11_O_4_	OH	OH	OH	OH	H	Skin	+	[55]
	Kaempferol	H	OH	OH	OH	H	H	Skin	100 ^d^ 1.71–1.96 ^a^ 2.75–12.1 ^a^ + + 1250.0 ^b^ 3982.9 ^e^	[23] [30] [53] [57] [55] [32] [56]
	Kaempferol-3-*O*-rutinoside	C_12_H_21_O_9_	OH	OH	OH	H	H	Hull	+	[6]
								Skin	+ + 12.80–31.80 ^a^ 5.26–40.7 ^a^ + + 22,949.3 ^b^ 10,829.9 ^e^	[58,6] [30] [53] [57] [55] [32] [56]
***Flavonoids***										
**Flavonols**		R_3_	R_5_	R_7_	R_4’_	R_5’_	R_6’_			
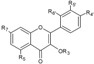	Kaempferol-3-*O*-glucoside	C_6_H_11_O_5_	OH	OH	OH	H	H	Hull	+	[6]
								Skin	+ + 39.0 ^d^ + 1.65 ^a^ 14.2 ^a^ + 39,012.3 ^b^ 4080.9 ^e^	[28] [58,23] [6] [30] [53] [57] [32] [56]
	Isorhamnetin	H	OH	OH	OH	CH_3_O	H	Hull	+	[6]
								Skin	47.2 ^d^ + 4.19–4.87 ^a^ 5.92–16.0 ^a^ + + 4551.9 ^b^	[23] [6] [30] [53] [57] [55] [32]
	Isorhamnetin-3-*O*-glucoside	C_6_H_11_O_5_	OH	OH	OH	CH_3_O	H	Hull	+	[6]
								Skin	+ + 8.85–15.60 ^a^ 5.02–15.3 ^a^ + 16,940.9 ^b^ 461.3 ^e^	[58] [6] [30] [53] [57] [32] [56]
***Flavonoids***										
**Flavonols**		R_3_	R_5_	R_7_	R_4’_	R_5’_	R_6’_			
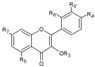	Isorhamnetin-3-*O*-rutinoside	C_12_H_21_O_9_	OH	OH	OH	CH_3_O	H	Skin	+ 40.4 ^d^ 27.60–41.40 ^a^ 5.34–58.0 ^a^ + + 54,901.1 ^b^ 210.8 ^e^	[58] [23] [30] [53] [57] [55] [32] [56]
	Morin	H	OH	OH	OH	H	OH	Hull	+	[6]
								Skin	+	[6]
***Flavonoids***										
**Flavanones**		R_5_	R_7_	R_4’_	R_5’_					
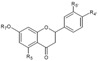	Naringenin-7-*O*-glucoside	OH	C_6_H_11_O_5_	OH	H			Skin	+ 16.1 ^d^ 6.84–22.10 ^a^ 2.34–25.9 ^a^ + + 14,288.1 ^b^	[28] [23] [30] [53] [57] [55] [32]
	Eriodictyol-7-*O*-glucoside	OH	C_6_H_11_O_5_	OH	OH			Skin	0.808–1.60 ^a^ 2.5–2.65 ^a^ + 3382.5 ^b^ 1309.2 ^e^	[30] [53] [55] [32] [56]

+ present; ^a^ µg/g of extract; ^b^µg/100 g; average of triplicate measurements; ^c^ mg/100 g of fresh weight; ^d^ % percent distribution from almonds; ^e^ µg/100 g fresh weight.

**Table 2:** Functional applications of almond by-products.

Destiny	Use	References
dairy industry	livestock feed	[80]
energy production	energy feedstock	[81]
agriculture	compost, soil ameliorants, mulch and/or fertilizers	[82,83,84,85,86,87]
particleboard manufacturing	wood-based composite	[2]
production of activated carbons	rich source in preparing activated carbons	[26,88,89]
nanopaper production	source of nanofibers	[90]
natural colorant	wool dyeing	[91,92]
